# The obesity paradox and incident cardiovascular disease: A population-based study

**DOI:** 10.1371/journal.pone.0188636

**Published:** 2017-12-07

**Authors:** Virginia W. Chang, Kenneth M. Langa, David Weir, Theodore J. Iwashyna

**Affiliations:** 1 Department of Social and Behavioral Sciences, College of Global Public Health, New York University, New York, New York, United States of America; 2 Department of Population Health, School of Medicine, New York University, New York, New York, United States of America; 3 Department of Medicine, University of Michigan Medical School, Ann Arbor, Michigan, United States of America; 4 Veterans Affairs Center for Clinical Management Research, Ann Arbor, Michigan, United States of America; 5 Institute for Healthcare Policy and Innovation, University of Michigan, Ann Arbor, Michigan, United States of America; 6 Institute for Social Research, University of Michigan, Ann Arbor, Michigan, United States of America; Tulane University School of Public Health and Tropical Medicine, UNITED STATES

## Abstract

**Background:**

Prior work suggests that obesity may confer a survival advantage among persons with cardiovascular disease (CVD). This obesity “paradox” is frequently studied in the context of prevalent disease, a stage in the disease process when confounding from illness-related weight loss and selective survival are especially problematic. Our objective was to examine the association of obesity with mortality among persons with incident CVD, where biases are potentially reduced, and to compare these findings with those based on prevalent disease.

**Methods:**

We used data from the Health and Retirement Study, an ongoing, nationally representative longitudinal survey of U.S. adults age 50 years and older initiated in 1992 and linked to Medicare claims. Cox proportional hazard models were used to estimate the association between weight status and mortality among persons with specific CVD diagnoses. CVD diagnoses were established by self-reported survey data as well as Medicare claims. Prevalent disease models used concurrent weight status, and incident disease models used pre-diagnosis weight status.

**Results:**

We examined myocardial infarction, congestive heart failure, stroke, and ischemic heart disease. A strong and significant obesity paradox was consistently observed in prevalent disease models (hazard of death 18–36% lower for obese class I relative to normal weight), replicating prior findings. However, in incident disease models of the same conditions in the same dataset, there was no evidence of this survival benefit. Findings from models using survey- vs. claims-based diagnoses were largely consistent.

**Conclusion:**

We observed an obesity paradox in prevalent CVD, replicating prior findings in a population-based sample with longer-term follow-up. In incident CVD, however, we did not find evidence of a survival advantage for obesity. Our findings do not offer support for reevaluating clinical and public health guidelines in pursuit of a potential obesity paradox.

## Introduction

Though obesity is a well-known risk factor for cardiovascular disease (CVD) [1], a more recent and debated literature suggests that obesity may be associated with improved survival once CVD is established [2–6], a finding that is termed an “obesity paradox”. For example, among persons with chronic heart failure, a meta-analysis estimates that obesity is associated with a 33% lower risk of mortality relative to the normal weight category [6]. Risk reductions of 30–40% have also been reported among persons with various types of ischemic heart disease [3–5].

There are various physiologic pathways by which obesity could confer a protective effect. Obese patients have more catabolic reserve, and catabolic stress occurs in states such as heart failure and acute myocardial infarction (MI) [6, 7]. Other potential pathways relate to neurohormonal, inflammatory, and hemodynamic processes. These include obesity-related differences in tumor necrosis factor (TNF-α), interleukin-10 (IL-10), leptin, sympathetic nervous system responses, renin-angiotensin responses, the detoxification of lipopolysaccharides, and circulating atrial natriuretic peptides [6–8]. For example, higher levels of IL-10 among obese patients may attenuate deleterious inflammatory processes, and receptors that bind TNF-α in fat tissue could help to decrease the adverse effects of TNF-α.

Despite plausible physiologic explanations for an obesity paradox, prior findings may be confounded by disease-related weight loss. Disease can lead to both death and weight loss, and both are a sign of more severe disease. If the normal weight category contains people who have lost weight because of more severe disease, then the adverse effects of obesity on mortality are systematically underestimated [9, 10]. Weight loss can be unintentional, due to the disease process itself, or intentional, as people with more severe disease may be more motivated to lose weight. There is also a related, but distinct issue of survivor bias. This would occur if those who are both obese and have severe disease are more likely to die early, leaving behind a more robust obese population at the time of study entry. Both of these problems are reduced by examining persons at the time of an incident, as opposed to prevalent, diagnosis of CVD and using their pre-diagnosis weight. Most prior work examines prevalent CVD [4–6, 11–23], a stage in the disease process where these biases are accentuated and especially problematic. Among studies included in meta-analyses, 95% examine prevalent disease [3–6].

Our objective was to use nationally representative longitudinal data to examine the association between obesity and mortality among persons with incident CVD, where biases are potentially reduced, and to compare findings with those based on prevalent CVD. While prevalent disease models rely on concurrent weight status, we can use pre-diagnosis weight status in incident disease models. This is the first study to compare the use of prevalent to incident case modeling *in the same dataset*, which attenuates differences due to variation in data source. We examined specific types of types CVD (e.g., myocardial infarction, congestive heart failure, and stroke), and we cross-validated results by comparing diagnoses based on survey self-report to those based on Medicare claims. Like other recent studies [21, 23] we contribute to prior work by examining a population-based sample. Much prior work is based on post-hoc analyses of clinical trial data [11–15] or single-center studies of patients referred to a subspecialty clinic (e.g., transplant, cardiac rehabilitation) [16–19] or for cardiac testing and intervention [4, 20]. Clinical trials enroll highly selected samples, and those in a subspecialty clinic differ from the general population in disease severity and access to care. For example, patients presenting for transplant evaluation or cardiac rehabilitation are at a more advanced state of disease than most cases of heart failure in the community. The nature of care delivered in these settings is similarly selective. Less is known about how findings from such clinical data relate to the broader U.S. population and longer-term follow-up.

A potential obesity paradox among persons with CVD has important clinical and public health implications. If obesity actually improves survival in this population, CVD guidelines recommending weight loss for those who are obese may be having unintended, adverse consequences [24]. Furthermore, national guidelines for the general population have endorsed weight loss for those who are obese without distinguishing between those with and without conditions where obesity may in fact be protective [25]. In view of accepting the existence of an obesity paradox, some have recently proposed replacing the language of “paradox” with “cardiovascular obesity paradigm” to highlight its standing as an oft-repeated finding [26].

## Methods

### Data

We used the Health and Retirement Study (HRS), which is conducted by the University of Michigan and designed as a nationally representative panel survey of U.S. adults age 50 and over, with various cohorts entering at specific time points since 1992 [27]. Over 30,000 individuals have contributed over 175,000 interviews thus far. Interviews are conducted every two years on an array of health topics and include the use of proxies if respondents are cognitively impaired. We used ten waves of survey data from 1992–2010 with mortality follow-up through December 31^st^, 2012. The HRS determines death by family member report and linkage with the National Death Index. In addition to the survey component, the HRS is linked to Medicare claims, providing information on both inpatient and outpatient claims-based diagnoses. Many persons are not yet age 65 (i.e., Medicare-eligible) when they enter the HRS, but become age-eligible over time.

### Prevalent and incident diagnoses

We examined the association of weight status with mortality among persons with specific types of CVD. First, we targeted persons with a prevalent diagnosis and examined whether findings from prior work suggesting a paradox are replicated. Second, we targeted incident diagnoses and used pre-diagnosis weight status to reduce bias from illness-related weight loss and selective survival.

#### Self-reported diagnoses

Prevalent disease was based on responses at study entry to individual questions about whether a doctor has ever told them that they have specific conditions. The HRS asks about congestive heart failure (CHF), myocardial infarction (“heart attack or myocardial infarction”), and stroke. The HRS also has a more general heart disease question on “heart attack, coronary heart disease, congestive heart failure, or other heart problems”. Though not as specific, we included analyses based on this category and labeled it “heart disease”. In all subsequent waves, participants are asked whether a condition has developed since the previous interview if not previously reported. We defined incident disease at a given wave using cases that were newly occurring.

#### Diagnoses based on Medicare claims

We used Medicare claims as an alternate method for identifying diagnoses and repeated analyses to cross-validate between methods. Analyses using claims were limited to persons age 65 and older enrolled in fee-for-service Medicare. As above, we identified CHF, MI, and stroke. In addition, we identified ischemic heart disease (IHD) to broaden the analysis beyond MI alone. Having an MI or CHF can be consequences of IHD, but many with IHD have not had an MI and do not have CHF. Prevalent diagnoses were identified by searching for claims within the first available two-year claims window using previously validated and conventional algorithms [28–33]. For persons without claims in this conventionally used “washout” period, incident diagnoses were identified by searching for subsequent claims. Incident diagnoses were timed by the date of the first qualifying claim. We used Medicare data from the denominator, Medicare Provider Analysis and Review, outpatient, carrier, and home healthcare files.

### Weight status and covariates

Body mass index (BMI: weight [kg]/height [m^2^]) from respondent-reported height and weight was categorized using standard guidelines to allow comparison with prior work: underweight (BMI <18.5), normal (BMI = 18.5–24.9), overweight (BMI = 25.0–29.9), class I obese (30.0–34.9), and class II/III obese (BMI ≥ 35.0) [34]. Covariates included sex, race/ethnicity, marital status, education, household income, household wealth, smoking status, self-rated health, and cohort. Age was the time scale in survival models. Household income and wealth were modeled as continuous variables adjusted for skew and inflation. Income was log transformed, and wealth was transformed with an inverse hyperbolic sine function. Wealth measured total household assets accounting for debts and included the value of real estate savings, retirement accounts, and investments. Other covariates were modeled with indicators for the categories shown in **Table 1**. The proportion dropped for missing data in any given model was less than 3%.

**Table 1:** Baseline characteristics.

		% or Mean (Interquartile Range)			
Variable		Overall Sample		Prevalent CVD	
N		30,529		5,870	
BMI Category (%)					
	Underweight (< 18.5)	1.9		3.1	
	Normal (18.5–24.9)	35.6		34.7	
	Overweight (25.0–29.9)	39.0		38.2	
	Class I obese (30.0–34.9)	15.9		15.9	
	Class II/III obese (≥ 35.0)	7.5		8.1	
Age (years)		62.0	(53.3–71.7)	68.5	(57.9–77.0)
Female (%)		56.4		49.1	
Race/ethnicity (%)					
	Non-Hispanic white	72.2		75.7	
	Non-Hispanic black	16.1		16.2	
	Hispanic	9.3		6.1	
	Other	2.4		2.0	
Married/partnered (%)		72.2		64.3	
Education (%)					
	< High school	28.5		38.3	
	GED/high school graduate	34.0		32.6	
	Some college	20.2		17.8	
	College graduate	17.4		11.4	
Household income ($1000)		73.9	(23.1–90.4)	53.8	(16.7–62.7)
Household wealth ($1000)		337.1	(38.4–351.4)	261.1	(16.6–284.6)
Smoking status (%)					
	Never	41.2		36.0	
	Former	37.4		45.7	
	Light (< 1 pack per day)	9.7		8.5	
	Moderate (1 to < 2 packs)	9.3		7.7	
	Heavy (≥ 2 packs)	2.4		2.2	
Self-rated health fair/poor (%)		27.4		56.1	
Dead (%)		39.4		67.3	
Follow-up duration (years)		11.8	(6.6–18.6)	9.0	(3.8–13.8)

Prevalent cardiovascular disease (CVD) = persons with a prevalent diagnosis of myocardial infarction, congestive heart failure, stroke, or a more general heart disease category on self-reported survey data. Household income and wealth are in 2012 dollars (end of follow-up).

### Statistical analyses

We used Cox proportional hazard models to examine the association between weight status and mortality with age in months as the time scale. Respondents who were alive at the end of the study period were censored, and proportionality assumptions were checked with Schoenfeld residuals. BMI categories were modeled with indicator variables, and all models adjusted for the covariates noted above. In order to focus our adjustment on confounding of the BMI variable, time-varying covariates such as marital status, measures of socioeconomic status, smoking, and self-rated health were assessed at the same wave as BMI in all of our models. As the HRS includes spouses, all models adjusted for household clustering with a robust variance estimator.

We ran separate models for the sample of persons with each of condition of interest. First, we modeled survival among persons with prevalent disease, replicating the structure of much prior work in our sample. In models using a survey-based diagnosis, BMI was assessed at the first HRS interview, which was the same wave used to establish preexisting disease and a prevalent diagnosis. When using a claims-based prevalent diagnosis, BMI was assessed at the first interview following the initial two-year claims window. Second, we modeled survival among those with an incident diagnosis and used pre-diagnosis weight status. We compared these models to those based on prevalent diagnoses to assess potential confounding from disease-related weight loss and survivor bias in the context of prevalent disease. In models using a survey-based incident diagnosis, BMI was assessed at the wave preceding the wave of diagnosis. When using a claims-based incident diagnosis, BMI was taken from the most recent interview that precedes the diagnosis date by at least one year. All analyses were conducted using STATA version 13.1 statistical software. The New York University Committee on Activities Involving Human Subjects determined that analysis of the public-use HRS data for this project is not considered research with human subjects. Analysis of the HRS-Medicare linked data used for this project was approved by the University of Michigan Medical School Institutional Review Board. HRS respondents provided oral informed consent at enrollment and for Medicare linkage.

## Results

**Table 1** shows descriptive characteristics at the initial interview for the overall HRS sample and persons with a self-reported diagnosis for any of the CVD outcomes. Relative to the overall sample, persons with prevalent CVD had a higher frequency of underweight and obese class II/III. They were also older and more likely to be male, not married/partnered, have lower socioeconomic status, report fair/poor health, and die during the study period.

**Table 2** presents relative hazards of mortality for various BMI categories among those with CVD diagnoses based on *self-reported data*. Normal weight is the reference category. Looking first at the prevalent cases (top panel of table), obese class I had a significantly lower hazard of mortality relative to normal weight. The estimated hazard reductions were 29% [95% confidence interval: 14%-42%] for MI, 36% [15%-51%] for CHF, 20% [3%-34%] for stroke, and 18% [8%-27%] for the more general category of heart disease. These findings suggest evidence of an obesity paradox, and the magnitude of the estimates is in the range of those previously reported for CVD. Overweight also showed lower mortality in some conditions. In the models for MI, CHF, and stroke, adjusting for the presence of the other two conditions resulted in minimal change to the estimates. For example, the hazard ratios (HRs) for obese class I in these models were 0.72 [0.59–0.87] for MI, 0.66 [0.50–0.87] for CHF, and 0.80 [0.66–0.97] for stroke.

**Table 2:** Hazard ratios for mortality, cases based on self-reported survey data.

**Survey Prevalent Cases**								
**BMI Categories**	**MI**	** **	**CHF**	** **	**Stroke**	** **	**Heart Disease**	
Underweight	2.33***	(1.52–3.56)	1.96**	(1.19–3.23)	1.30	(0.90–1.87)	1.65***	(1.34–2.02)
Normal	1.00	(REF)	1.00	(REF)	1.00	(REF)	1.00	(REF)
Overweight	0.85*	(0.74–0.98)	0.82	(0.66–1.03)	0.94	(0.82–1.07)	0.88**	(0.81–0.96)
Obese class I	0.71***	(0.58–0.86)	0.64**	(0.49–0.85)	0.80*	(0.66–0.97)	0.82**	(0.73–0.92)
Obese class II/III	0.93	(0.72–1.19)	0.81	(0.60–1.09)	0.99	(0.78–1.27)	1.06	(0.91–1.23)
N	1713		798		1545		4820	
**Survey Incident Cases**								
**BMI Categories**	**MI**	** **	**CHF**	** **	**Stroke**	** **	**Heart Disease**	
Underweight	2.19***	(1.51–3.17)	2.06***	(1.46–2.91)	1.52*	(1.08–2.12)	1.75***	(1.32–2.33)
Normal	1.00	(REF)	1.00	(REF)	1.00	(REF)	1.00	(REF)
Overweight	0.79**	(0.69–0.91)	0.99	(0.86–1.12)	0.90	(0.80–1.01)	0.87**	(0.79–0.96)
Obese class I	0.97	(0.81–1.16)	0.85	(0.72–1.01)	0.98	(0.83–1.14)	0.91	(0.80–1.04)
Obese class II/III	1.08	(0.85–1.37)	0.94	(0.77–1.16)	1.05	(0.84–1.31)	1.22*	(1.04–1.44)
N	2237		2121		2684		5048	

Underweight (BMI <18.5), normal (BMI = 18.5–24.9), overweight (BMI = 25.0–29.9), obese class I (30.0–34.9), obese class II/III (BMI≥ 35). MI = myocardial infarction, CHF = congestive heart failure. Heart disease refers to a more general question in the survey on “heart attack, coronary heart disease, congestive heart failure, or other heart problems.” Numbers in parentheses are 95% confidence intervals. All models adjust for sex, race/ethnicity, marital status, cohort, education, household income, household wealth, smoking status, and self-rated health.

*p < .05

**p < .01

***p < .001

The bottom panel in **Table 2** shows models using incident cases and pre-diagnosis weight for the same conditions. In each of the four conditions, the HR for obese class I was greatly attenuated (toward the null) compared to its estimate in the corresponding prevalent model, and in all cases it was no longer statistically significant despite larger sample sizes. Hence, there was no longer evidence of an obesity paradox when switching from prevalent to incident models in the same dataset. Overweight remained associated with lower mortality in MI and heart disease, and obese class II/III was significantly associated with higher mortality in heart disease.

**Table 3** presents cases based on *Medicare claims*, where diagnoses are based on health provider coding rather than respondent self-report. The number of cases identified via the two methods is not directly comparable because claims analyses were limited to persons age 65 and older. In addition to MI, CHF, and stroke, we were able to capture IHD, which is a diagnosis that most persons would not be able to self-report in a survey. Persons with IHD have coronary vessels at risk for an event, but they have not necessarily had such an event.

**Table 3:** Hazard ratios for mortality, cases based on Medicare claims data.

**Medicare Claims Prevalent Cases**								
**BMI Categories**	**MI**	** **	**CHF**	** **	**Stroke**	** **	**Ischemic Heart Disease**	
Underweight	1.85*	(1.10–3.12)	1.92***	(1.36–2.70)	1.27	(0.57–2.83)	1.37*	(1.04–1.82)
Normal	1.00	(REF)	1.00	(REF)	1.00	(REF)	1.00	(REF)
Overweight	0.79	(0.61–1.02)	0.86*	(0.75–0.99)	0.79*	(0.62–0.99)	0.87**	(0.79–0.95)
Obese class I	0.69	(0.45–1.05)	0.65***	(0.53–0.79)	0.66*	(0.48–0.92)	0.82**	(0.72–0.93)
Obese class II/III	0.55	(0.29–1.05)	0.76*	(0.61–0.94)	1.28	(0.86–1.92)	0.98	(0.83–1.17)
N	402		1217		490		3538	
**Medicare Claims Incident Cases**								
**BMI Categories**	**MI**	** **	**CHF**	** **	**Stroke**	** **	**Ischemic Heart Disease**	
Underweight	1.43**	(1.10–1.85)	1.36**	(1.13–1.63)	1.25	(0.86–1.82)	1.49***	(1.23–1.80)
Normal	1.00	(REF)	1.00	(REF)	1.00	(REF)	1.00	(REF)
Overweight	0.87*	(0.75–0.99)	0.90*	(0.83–0.97)	0.87*	(0.78–0.97)	0.90**	(0.83–0.97)
Obese class I	1.00	(0.82–1.21)	0.94	(0.84–1.05)	0.99	(0.85–1.16)	0.92	(0.81–1.03)
Obese class II/III	0.94	(0.69–1.28)	0.97	(0.83–1.14)	1.02	(0.80–1.29)	1.16	(0.99–1.37)
N	1148		2942		1660		4218	

Underweight (BMI <18.5), normal (BMI = 18.5–24.9), overweight (BMI = 25.0–29.9), obese class I (30.0–34.9), obese class II/III (BMI≥ 35). MI = myocardial infarction, CHF = congestive heart failure. Numbers in parentheses are 95% confidence intervals. All models adjust for sex, race/ethnicity, marital status, cohort, education, household income, household wealth, smoking status, and self-rated health.

*p < .05

**p < .01

***p < .001

The top panel of **Table 3** shows claims-based prevalent cases. The number of cases for MI and stroke were low relative to CHF and IHD because they are events that occur at distinct points in time, whereas the latter two persist. Events are less likely to occur than enduring states in any two-year window, which is the window conventionally used to establish prevalent disease in the claims. Here, the prevalent models again suggested a paradox for obese class I. The estimated hazard reductions were 31% [95% CI: -5%-55%] for MI, 35% [21%-47%] for CHF, 34% [8%-52%] for stroke, and 18% [7%-28%] for IHD. The finding of a paradox for obese class I with estimates of similar magnitude in both survey- and claims-based models lends confidence to each method of disease ascertainment. Though the estimate for obese class I in MI was only 10% significant in the claims model, the HR (0.69) was similar to the HR in the survey-based model (0.71), and the sample size was much smaller.

The bottom panel of **Table 3** shows the results for claims-based incident cases and pre-diagnosis weight status. The HRs for obese class I were greatly attenuated (toward the null) relative to the prevalent case models and lost significance despite larger sample sizes. In keeping with the pattern observed for survey-based cases (in **Table 2**), there was no evidence of an obesity paradox when switching to models based on incident diagnoses and pre-diagnosis weight. Reduced hazards for obese class II/III in prevalent MI and prevalent CHF were also attenuated toward the null and not significant. Overweight remained associated with lower mortality.

In **Table 4**, we further assessed potential weight loss confounding by excluding deaths within the first three years of diagnosis in the incident models, as early or more precipitous deaths can reflect greater disease severity. Here, there was no longer a significant protective effect for overweight, and all estimates for overweight and both categories of obesity were higher, with several showing significantly higher mortality relative to normal weight. Hence, the protective effect observed for overweight in some of our incident models may have been due to residual confounding by disease severity. Though dropping early deaths also increased HRs in prevalent models, many estimates continued to suggest an obesity paradox in the setting of prevalent disease.

**Table 4:** Hazard ratios for mortality, incident cases excluding deaths in the first 3 years.

**Survey Incident Cases**								
**BMI Categories**	**MI**	** **	**CHF**	** **	**Stroke**	** **	**Heart Disease**	
Underweight	1.73*	(1.09–2.73)	1.87**	(1.19–2.95)	1.14	(0.72–1.81)	1.24	(0.87–1.77)
Normal	1.00	(REF)	1.00	(REF)	1.00	(REF)	1.00	(REF)
Overweight	0.88	(0.74–1.03)	1.28**	(1.09–1.51)	1.05	(0.91–1.20)	1.01	(0.90–1.14)
Obese class I	1.24*	(1.01–1.52)	1.14	(0.93–1.40)	1.11	(0.92–1.33)	1.17*	(1.00–1.36)
Obese class II/III	1.36*	(1.04–1.77)	1.43**	(1.12–1.84)	1.36*	(1.04–1.78)	1.66***	(1.38–1.99)
N	1777		1453		1975		4149	
**Medicare Claims Incident Cases**								
**BMI Categories**	**MI**	** **	**CHF**	** **	**Stroke**	** **	**Ischemic Heart Disease**	
Underweight	1.47	(0.93–2.34)	1.15	(0.83–1.60)	0.84	(0.40–1.74)	1.20	(0.90–1.61)
Normal	1.00	(REF)	1.00	(REF)	1.00	(REF)	1.00	(REF)
Overweight	1.00	(0.80–1.24)	1.02	(0.91–1.15)	1.03	(0.88–1.21)	0.98	(0.89–1.09)
Obese class I	1.36	(0.98–1.87)	1.19*	(1.01–1.41)	1.14	(0.91–1.44)	1.03	(0.88–1.20)
Obese class II/III	1.49	(0.89–2.50)	1.42**	(1.13–1.78)	1.52*	(1.09–2.13)	1.53***	(1.25–1.88)
N	586		1657		920		3126	

Underweight (BMI <18.5), normal (BMI = 18.5–24.9), overweight (BMI = 25.0–29.9), obese class I (30.0–34.9), obese class II/III (BMI≥ 35). MI = myocardial infarction, CHF = congestive heart failure. Heart disease refers to a more general question in the survey on “heart attack, coronary heart disease, congestive heart failure, or other heart problems.” Numbers in parentheses are 95% confidence intervals. All models adjust for sex, race/ethnicity, marital status, cohort, education, household income, household wealth, smoking status, and self-rated health.

*p < .05

**p < .01

***p < .001

Allowing for interactions with sex did not significantly change our results. We also conducted sensitivity analyses where incident cases from the survey were re-examined at a subsequent wave as surviving prevalent cases (**S1 Table**). In our main models, incident and prevalent cases were, by definition, not the same people. Here, those who survived and interviewed at least two waves after an incident diagnosis wave were re-analyzed using this later wave–and the BMI at that wave–as the baseline. In this approximation of a prevalent case analysis, we again observed a lower risk of mortality for obese class I vs. normal weight for each diagnosis, confirming that timing in the disease process is an important factor.

## Discussion

Prior work suggests that among persons with CVD, obese persons, and particularly those with mild obesity, may have a lower risk of mortality relative to those at normal weight. We examined prevalent diagnoses as well as incident diagnoses and pre-diagnosis weight for specific types of CVD. In all conditions we found a consistent pattern: a strong and significant obesity paradox in prevalent models with substantive attenuation and absence of support in incident models, despite larger sample sizes. Furthermore, the findings between the self-reported vs claims-based models were largely consistent, lending confidence to each approach for ascertaining diagnoses of CVD.

Prevalent disease is inescapably plagued by disease-related weight loss and selective survival, both of which can bias findings towards a paradox. The absence of support for a paradox when switching from prevalent to incident cases and pre-diagnosis weight suggests that prevalent models are likely biased by such factors. These findings are in keeping with work on prevalent CVD in cross-sectional data finding no evidence for an obesity paradox when the reference category is restricted to those who have always been normal weight based on recalled data [21]. Given the widespread use of prevalent case analyses in this literature, the extent of bias from disease-related confounding in prevalent disease may be underappreciated.

Restricting to incident disease is by no means a complete solution to disease-related weight loss, as there can be preclinical disease and weight loss prior to receiving a diagnosis. However, we expect such confounding to be meaningfully reduced relative to sampling on prevalent cases where the disease has had more time to progress and we only see survivors at a given time point. Even with potential residual confounding, we found no evidence to suggest an obesity paradox when examining incident CVD and pre-diagnosis weight. This simple and straightforward adjustment was enough to lose support for an obesity paradox, despite larger sample sizes. Our conclusions are in keeping with some [35, 36], but not all [37, 38], prior work on incident MI. Prior work suggesting a protective effect, however, is limited by the inclusion of underweight in the referent group and a lack of pre-diagnosis weight [37, 38].

Another approach to limit weight loss confounding is excluding early deaths. This and other exclusions, however, typically result in dropping a very large proportion of deaths and selects on other unobserved characteristics [39]. Some have argued these deletions may increase rather than reduce bias [39]. Bearing potential limitations in mind, when we dropped early deaths, all estimates further moved in the positive direction, suggesting that both classes of obesity (and overweight in CHF) may be associated with increased mortality in CVD. Further restricting the sample to never smokers did not meaningfully change results. While obesity may, in fact, reduce survival in CVD, our primary objective was to examine whether it might be *protective*, and, without dropping a large proportion of deaths, we found that this claim was not supported.

Another explanation for the obesity paradox relates to a form of selection known as collider bias, though there are inconsistent findings with respect to plausibility [23, 40]. Because obesity increases the risk of CVD, non-obese persons with CVD are more likely to have other risk factors. If one or more of these other factors has a stronger effect on mortality than obesity itself, it can induce a spurious, inverse association between obesity and mortality when conditioning on CVD. As collider bias would be an issue regardless of whether one is conditioning on incident or prevalent CVD, it does not seem to be an obvious explanation for the pattern of changes that we observe. Importantly, collider bias does not preclude the existence, or co-existence, of other forms of bias such as weight loss confounding and reverse causality [23], which also merit investigation and are the focus of this study. As collider bias relates to a potential myriad of unobserved factors that cannot be accounted for, we cannot rule out its potential influence, and it is possible that the true estimates for obesity are higher. Accepting this potential underlying influence, however, we did not have evidence for a protective effect of obesity in incident case models.

This study has limitations. First, BMI was based on self-reported height and weight. Prior work on the HRS finds that misclassification from the use of these self-reported values does not significantly bias estimates for the association between obesity and mortality in these data [41]. Other datasets with measured BMI are either cross-sectional or not nationally representative. Importantly, the changes we observed in comparing prevalent to incident disease are unlikely a product of using self-reported BMI.

Second, BMI is an imperfect measure of adiposity. The use of BMI, however, allows for a comparison of our findings to prior work showing a BMI-defined obesity paradox. Moreover, the examination of weight status—as defined by BMI—is of practical relevance. For persons with CVD, national guidelines have advocated weight loss for those who are obese, as defined by BMI. As such, it is important to know whether BMI-defined obesity is actually associated with improved survival. In this sense, one primary goal is not to study body fat per se, but rather the central measure used in clinical guidelines. BMI is also the only measure routinely obtained in the clinical setting. Finally, this limitation of BMI is typically raised as a factor can bias findings *toward an obesity paradox*, rather than away from a paradox, because lower weight may be picking up lower muscle mass, which can be associated with lower survival. As we conclude that there was not evidence of a paradox, this would lend a conservative bias to our conclusions.

Third, there are limitations to our ascertainment of CVD. The self-reported data risk a poor understanding of formal diagnoses, recall error, and missed incident cases from death between surveys. While the claims data are subject to coding error, they offer assessments by clinicians, and fewer incident cases are missed due to continuous data capture. An advantage of the self-report data is that it includes persons under age 65. We view these approaches as complementary, and the consistency of results between them lends strength to our conclusions.

We found that the study of persons with prevalent CVD shows an obesity paradox, replicating findings from selected, clinical samples in a population-based sample with longer-term follow-up. However, prevalent disease-related weight loss at the baseline survey is likely a significant confounder in this setting. When switching to incident diagnoses of the same conditions and pre-diagnosis weight in the same dataset, we did not find evidence of a survival advantage for obesity. Our findings do not offer support for reevaluating current clinical and public health guidelines in pursuit of a potential obesity paradox.

## Supporting information

S1 Table Hazard ratios for mortality, simulated survey prevalent cases. (PDF)
