# Supplementary material for: The obesity paradox and incident cardiovascular disease: A population-based study
Source: PLoS One. 2017 Dec 7;12(12):e0188636. doi: 10.1371/journal.pone.0188636 (PMC5720539; doi:10.1371/journal.pone.0188636)
Supplement: S1 Table — (PDF) [file pone.0188636.s001.pdf]

S1 Table. Hazard Ratios for Mortality, Simulated Survey Prevalent Cases

BMI Categories	MI		CHF		Stroke		Heart Disease	
Underweight	1.50	(0.90-2.51)	1.67*	(1.10-2.54)	1.57*	(1.02-2.44)	1.63**	(1.18-2.25)
Normal	1.00	(REF)	1.00	(REF)	1.00	(REF)	1.00	(REF)
Overweight	0.66***	(0.54-0.81)	0.85	(0.67-1.08)	0.80*	(0.67-0.96)	0.73***	(0.63-0.85)
Obese class I	0.62**	(0.47-0.81)	0.69*	(0.53-0.91)	0.82	(0.64-1.05)	0.66***	(0.54-0.80)
Obese class II/III	0.82	(0.61-1.11)	0.79	(0.58-1.07)	0.86	(0.61-1.21)	0.95	(0.76-1.19)
N	1226		890		1228		2702	

Notes: The sample for each diagnosis is composed persons with a survey-based incident diagnosis who survived and interviewed at least two waves after the incident diagnosis wave. The baseline for analysis is this subsequent wave. Underweight (BMI <18.5), normal (BMI=18.5-24.9), overweight (BMI=25.0-29.9), obese class I (30.0-34.9), obese class II/III (BMI≥ 35). MI=myocardial infarction, CHF=congestive heart failure. Heart disease refers to a more general question in the survey on "heart attack, coronary heart disease, congestive heart failure, or other heart problems." Numbers in parentheses are 95% confidence intervals. All models adjust for sex, race/ethnicity, marital status, cohort, education, household income, household wealth, smoking status, and self-rated health.

*$p < .05$, **$p < .01$, ***$p < .001$
